# Simultaneous Removal and Recovery of Metal Ions and Dyes from Wastewater through Montmorillonite Clay Mineral

**DOI:** 10.3390/nano9121699

**Published:** 2019-11-28

**Authors:** Filippo Parisi, Giuseppe Lazzara, Marcello Merli, Stefana Milioto, Francesco Princivalle, Luciana Sciascia

**Affiliations:** 1 Dipartimento di Fisica e Chimica, Università degli Studi di Palermo, Viale delle Scienze, Ed. 17, 90128 Palermo, Italy; giuseppe.lazzara@unipa.it (G.L.); stefana.milioto@unipa.it (S.M.); 2 Dipartimento di Scienze della Terra e del Mare, Università degli Studi di Palermo, Via Archirafi, 22, 90123 Palermo, Italy; marcello.merli@unipa.it (M.M.); luciana.sciascia@unipa.it (L.S.); 3 Dipartimento di Matematica e Geoscienze, Università degli Studi di Trieste, Via Weiss, 1, 34128 Trieste, Italy; princiva@units.it

**Keywords:** Montmorillonite, adsorption, wastewaters, metal ions, dyes

## Abstract

The main objective of this work was to evaluate the potential of Montmorillonite nanoclay (Mt), readily and inexpensively available, for the simultaneous adsorption (and removal) of two classes of pollutants: metal ions and dyes. The attention was focused on two “model” pollutants: Ce(III) and crystal violet (CV). The choice is due to the fact that they are widespread in wastewaters of various origins. These characteristics, together with their effect on human health, make them ideal for studies on water remediation. Moreover, when separated from wastewater, they can be recycled individually in industrial production with no or simple treatment. Clay/pollutant hybrids were prepared under different pH conditions and characterized through the construction of the adsorption isotherms and powder X-ray diffraction. The adsorption behavior of the two contaminants was revealed to be significantly different: the Langmuir model reproduces the adsorption isotherm of Ce(III) better, thus indicating that the clay offers a unique adsorption site to the metal ions, while the Freundlich model proved to be the most reliable for the uptake of CV which implies heterogeneity of adsorption sites. Moreover, metal ions do not adsorb at all under acidic conditions, whereas the dye is able to adsorb under all the investigated conditions. The possibility to modulate the adsorption features by simply changing the pH conditions was successfully employed to develop an efficient protocol for the removal and separation of the different components from aqueous solutions mimicking wastewaters.

## 1. Introduction

The disposal of wastewaters from various origins represents a serious environmental issue due to the simultaneous presence of different types of pollutants [1,2,3,4]. Dyes and metals are widely used and often jointly released in large quantities from industrial activities such as dye manufacturing, the textile and leather tannery industries, pulp and paper processing, battery production [5,6,7,8,9,10,11].

Owing to their toxic potential and their recalcitrant capacity, discharge of metal ions and dyes effluents can cause potential hazards to environment and human health [12,13,14,15,16,17,18,19,20,21,22,23,24].

All conventional methods applied for the treatment of dyes and/or heavy metals [25,26,27,28,29,30,31,32] have peculiar limitations related to cost, efficiency and operational difficulties [11,33,34,35,36]. Among them, adsorption was revealed as one of the most effective methods due to its simple operation, versatility, high-treatment efficiency and low cost, and it is therefore widely applied for the treatment of wastewaters [37,38,39,40,41,42,43,44,45,46,47,48]. 

Several kinds of natural or chemically modified materials including activated carbons, carbon nanotubes, zeolites and clays [8,49,50,51,52,53,54,55,56,57,58], were investigated to remove contaminants from effluents. In recent years, there has been growing interest in clay minerals which are green, inexpensive and effective adsorption substrates [5,59,60,61,62,63,64,65].

Aside from their large surface area, the adsorption properties of clay minerals are mostly related to the negative charges generated by isomorphic substitutions. Generally, these negative charges are neutralized by exchangeable ions thus allowing the adsorption of positively charged cations through cation exchange processes. For these reasons clay minerals display a strong attraction to cationic species, such as dyes and metal ions [55,66,67,68,69,70]. 

Specifically, montmorillonite (Mt), whose structure consists of ~1 nm thick alluminosilicate layers, was largely employed in remediation due to its noteworthy properties, including large specific surface area and presence of nano-pores, high cations exchange capacity, presence of several types of active sites on the surface, easy availability, eco-friendliness and non-toxicity [71,72,73,74,75,76,77,78]. 

In this context is inserted the present work where Mt mineral clay was employed for the treatment of water samples containing two “model” pollutants. The organic dye crystal violet (CV) and the Ce(III) metal ions were chosen as models for dye metals because they are both in the cationic form in a wide range of pH, they are widespread in wastewaters of various origin, and have a toxic effect on human health, making them ideal for a study on water remediation. Moreover, when separated from wastewater, they can be recycled individually in industrial production [79].

In more detail, crystal violet is largely employed in textile and paper industries, in veterinary pharmacology and in bacteriology as Gram stain [79]. In spite of the great range of applications, crystal violet is a mutagen, carcinogenic and mitotic poison [80,81] and therefore the disposal of effluents is an important environmental issue. 

As for the Ce(III) species, it represents the most abundant element of rare earth metals and has several applications in engineering, agriculture, catalysis, nuclear energy, metallurgy, pharmaceutical, and removal from radioactive wastes [82,83,84,85,86,87,88]. Cerium compounds are considered to be moderately toxic [89,90,91,92] with their tendency to accumulate in the bones, liver, heart and lung and to react with enzyme and phospholipids [93,94]. Moreover, cerium in forms of nitrate and chloride could induce chromosomal breaks [95] and intensifying the cardiac effects of magnesium deficiency [96], respectively. Due to the toxic effect and the simultaneous technological importance of Ce(III), separation and recovery of these metal ions from effluents has a significant environmental and economic impact. 

The removal of both Ce(III) and CV from aqueous solutions through adsorption onto various substrates, including clay minerals, was investigated by various authors [20,24,34,81,83,97,98]. However, a systematic study aimed to develop an efficient procedure for their simultaneous removal and separation is still lacking. 

In the light of the above considerations, the aim of this work was to exploit the adsorption features of Mt clay for the treatment of aqueous solutions containing crystal violet and Cerium(III) as models for dyes and metals. Although different works concerning multicomponent adsorption were performed [99,100] in order to better clarify the adsorption mechanism and to propose a separation protocol, the adsorption behavior of the two contaminants separately was investigated here. Batch adsorption experiments were performed under different pH conditions and the adsorption isotherms were constructed in order to elucidate the adsorption mechanism and establish the nature of the interactions. The sites of interactions of the clay surface were proposed on the basis of the XRD results. Then, based on the information obtained, two different procedures were developed to remove simultaneously and separate the metal ions and dyes from the effluent.

## 2. Materials and Methods 

### 2.1. Materials

All the reactants, i.e., K10-Montmorillonite (Mt), hydrochloric acid (HCl), sodium hydroxide (NaOH) standard solutions, Ce(III) nitrate hexahydrate (Ce(III)) and crystal violet (CV, C_25_H_30_ClN_3_, Mw = 407.99 g mol^−1^, water solubility = 50 mg mL^−1^ at 27 °C, K_ow_ = 0.51) were purchased from Sigma Aldrich and used as received. The structural formula of K10-Mt is reported as follows: 

(K_0.25_Na_0.118_Ca_0.022_)(Al_1.06_Fe_0.206_Mg_0.166_)(Si_7.39_Al_0.61_)O_20_(OH)_4._

The BET (Brunauer-Emmett-Teller) surface area is 220 m^2^/g, the CEC is 119 meq/100 g, the total pore volume is 0.3 cm^3^/g and the average pore size is 6.25 nm. Pore size distribution (PSD) curves reveal that 80% of pores have a diameter <25 nm with a peak at 4.04 nm [101].

Zeta potential measurements reported in literature for K10-Montmorillonite [78] showed that it does not present isoelectric point, being the clay surface negatively charged at all pH values. 

Deionized water from reverse osmosis (Elga, model Option 3), having a specific resistance higher than 1 MΩ cm, was used to prepare all solutions.

### 2.2. Samples Preparation

Aqueous HCl and NaOH solutions at the desired pH were prepared by proper dilution of the corresponding standard solution. 

Pollutant stock solutions and Mt suspensions were prepared by weighing the proper amounts of the components and dissolving them with the aqueous solutions at the required pH, according to the procedure already reported in the literature [72]. When necessary the pH of the aqueous solutions/dispersions were adjusted to the desired value by adding microvolumes of either HCl or NaOH standard solution. The clay dispersions were stirred for about 2 h before use. 

In order to construct the adsorption isotherms, appropriate aliquots of the metal or dye solutions were added to the Mt dispersion at room temperature (25 °C). The pollutant concentrations were changed in the range from (2.0 ± 0.1) × 10^−4^ to (4.0 ± 0.2) × 10^−3^ g dm^−3^, while the amount of Mt was kept constant at 0.40 ± 0.02 g dm^−3^. The mixture was stirred at 100 rpm for 24 h, a stirring time which ensures that the adsorption processes reaches the equilibrium, as demonstrated by preliminary kinetic experiments. At the end of the adsorption process the pH of the obtained dispersions was checked. No significant changes were observed. The dispersion was then centrifuged 1 hour at 10,000 rpm by means of a Centra MP4R IEC centrifuge (Thermo Fisher Scientific, Waltham, MA, USA). The supernatant was separated from the solid, which was air-dried at room temperature, crushed in an agate mortar and employed for X-ray diffraction (XRD) characterization. 

The gathered supernatants were spectrophotometrically analyzed by registering the spectra of the aqueous pollutant solutions in the wavelength range 200–700 nm with a diode-array S600 spectrophotometer (Analytic Jena, Jena, Thuringia, Germany) equipped with thermostated compartments for 1 cm × 1 cm × 5 cm cuvettes and an appropriate magnetic stirring apparatus. Triplicate experiments were performed and the results are reported as the average value of each single measurement. 

The molar adsorption coefficient values (ε) of CV and Ce(III) at two different pH conditions were determined by constructing the calibration curves (Table 1)

### 2.3. X-ray Diffraction (XRD) Characterization

Powder X-ray diffractometry measurements were performed for the Mt/contaminant hybrids and for the clay in the absence of additives. Samples were mounted on aluminum plates and the XRD patterns were acquired at room temperature with an STOE D500 (Siemens, Monaco, Germany) with Cu Kα radiation, λ = 1.5418 Å, generated at 40 kV and 20 mA, in the range of scattering angles 2θ = 4°–25° at the rate of 0.01°/s.

## 3. Results

### 3.1. Effect of pH Solution on Adsorption Efficiency

Since clay surface is negatively charged at all pH values [78], Mt surface is ideal for the uptake of cations.

In order to compare the amount of the two contaminants adsorbed onto the clay under different pH conditions (pH = 3.0, 5.0, 7.0, 9.0), preliminary experiments were performed by mixing 0.04 g dm^−3^ of CV or Ce(III) with 0.4 g dm^−3^ of Mt. The dispersion obtained was then stirred and centrifuged and the supernatants were analysed spectrophotometrically as already described. The results, expressed in terms of weight percentage, i.e (mass of adsorbed component/initial mass) × 100%, are summarized in Table 2.

Results are not reported for the highest value of pH (pH = 9.0), since it was observed that the stability of the clay suspension is strongly decreased at high pH, thus leading to low reproducibility of the experiments.

Despite the fact that both contaminants are in the cationic form at the investigated pH range [102,103,104], a marked difference in the adsorption behavior between the species is clearly evidenced from data in Table 2. In more detail, Ce(III) metal ions do not adsorb onto clay at acidic pH at all, while the dye is able to adsorb at the three investigated pH conditions. The higher pH favors significantly the uptake of the dye according to what is observed for instance in [76] for the adsorption of methyl green dye molecule onto Mt clay.

As widely reported in the literature [72,105,106] the uptake of cationic species onto Mt clay occurs through cationic exchange processes in the clay interlayer and electrostatic interactions with the permanent negative charges on the clay surface. Moreover, the effect of the pH-dependent charges has to be taken into account: the abundance of H^+^ ions at acidic pH, imparts a repulsive force toward the positively charged species, thus hampering their uptake. The results obtained in the present work seem to indicate that electrostatic repulsions are predominant in the case of the adsorption of Ce(III) metal ions and less influent in the case of the crystal violet.

### 3.2. Adsorption Isotherms

**Figure 1:**
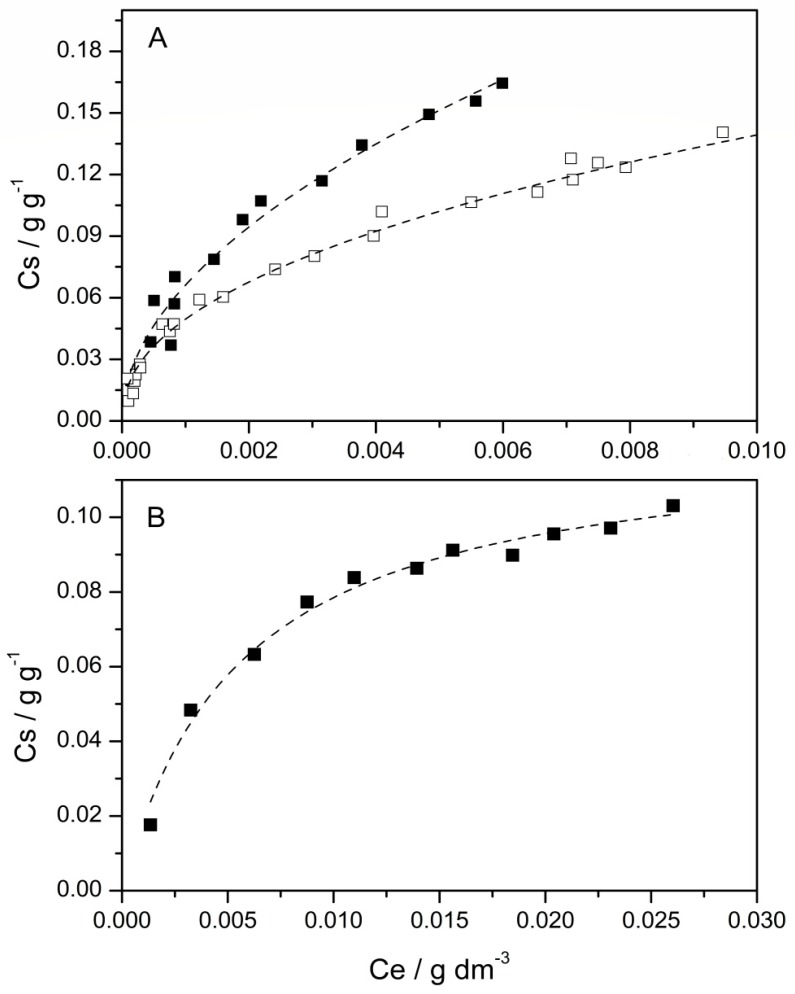
Adsorption isotherm of (**A**) CV and (**B**) Ce(III) onto Mt performed at pH = 7.0 (■) and pH = 3.0 (□). Lines correspond to the fit by Freundlich and Langmuir models, for CV and Ce(III) respectively.

The adsorption isotherms, where the equilibrium amount of pollutant adsorbed into the clay (Cs, g g^−1^) is plotted as a function of the equilibrium concentration in solution (Ce, g dm^−3^), are reported in Figure 1. In the light of the results reported in 3.1, two representative values of pH (pH 3.0 and pH = 7.0) were taken under consideration. It is worth underlining that, under the applied experimental conditions, no Ce(III) precipitation was observed.

As already observed the uptake of the dye is lower under the more acidic conditions, while the metal ions adsorb onto clay only at the higher pH value.

The following two models were used for fitting the adsorption isotherms:

- Langmuir isotherm:

Cs=qmKLCe1+KLCe

where *q^m^* and *K_L_* are Langmuir coefficients related to adsorption capacity and adsorption equilibrium constant respectively; 

- Freundlich isotherm model:

$$C_{s}=K_{F}C_{e}^{1/n}$$

where *K_F_* and *n* are Freundlich coefficients related to adsorption capacity and adsorption intensity, respectively.

The discrimination between the two models was performed by means of the statistical criteria described in [107] based on advanced statistical diagnostics and robust fitting techniques. The sorption parameters obtained and the most commonly applied statistics are collected in Table 3.

The Freundlich model proved to be the most reliable for the uptake of CV which implies heterogeneity of adsorption sites and/or formation of multilayers [108,109,110,111], while the Langmuir model better reproduces the adsorption isotherm of Ce(III), indicating that the clay offers a unique adsorption site to the metal ions. Adsorption isotherms reported in Figure 1 clearly evidences the higher adsorption capacity of CV with respect to Ce(III). However, since the two contaminants adsorb through different mechanisms, the adsorption parameters obtained are not suitable for comparison. 

As for the dye uptake, *K_F_* coefficient is higher at the higher pH, thus confirming that the excess of H ^+^ ions at the acidic pH hampers the CV adsorption. The Freundlich constant values n > 1.0 indicate the occurrence of favorable adsorption [78] and do not show significant variation with pH within the error bars. The obtained parameters are in line with those reported literature for the adsorption of CV onto K10-Montnorillonite [78,112]. Comparison with other adsorbents evidences the higher efficiency of Mt [33,113,114].

To the best of the authors’ knowledge, no data related to the adsorption of Ce(III) cations onto K10-Mt are available. The adsorption capacity values reported in literature for the uptake onto different supports [115,116,117] are of the same order of magnitude or lower than those obtained in the present work.

Comparable values of adsorption efficiency are achieved with the application of membrane separation processes, i.e., micro-, nano- or ultra-filration or reverse osmosis [118,119,120] in the removal of both classes of contaminants. However, although quite effective, these methods are characterized by elevated maintenance and operation costs and high energy requirements [118,121] which make them unsuitable, especially for small and medium industries. 

**Figure 2:**
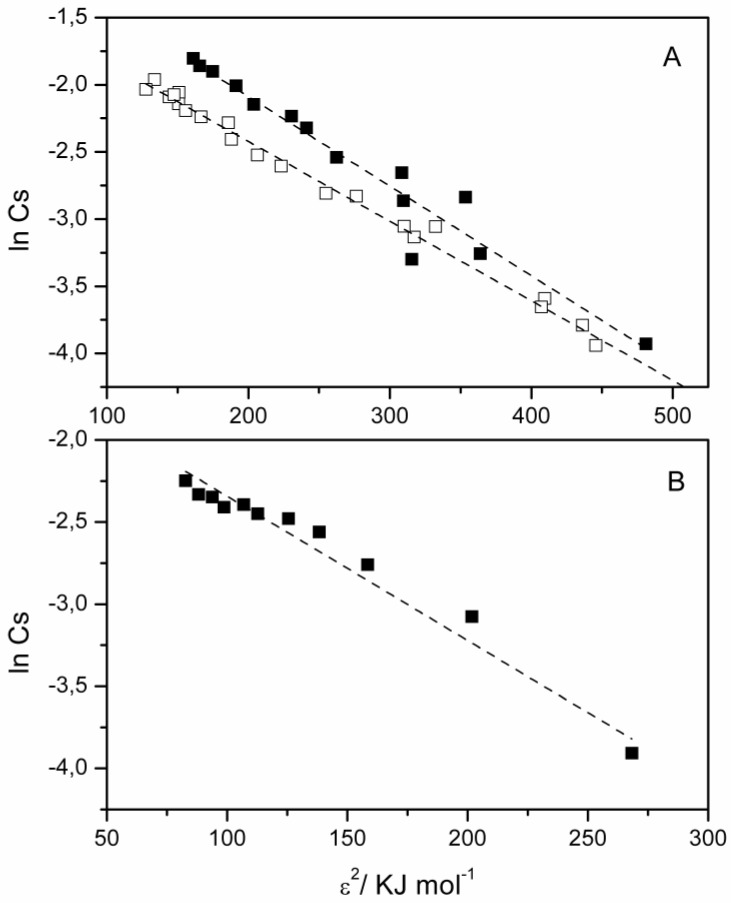
Dubinin–Radushkevich (DR) adsorption isotherms of (**A**) CV and (**B**) Ce(III) onto Mt performed at pH = 7.0 (■) and pH = 3.0 (□); line corresponds to the fit by DR equation.

Information about the kind of energy that governs the adsorption process was obtained by applying the Dubinin–Radushkevich (DR) equation (Figure 2):

ln*C_s_* = lnXm−kε^2^ (1)

where

ε = RT ln (1 + 1/*C_e_*) (2)

is the Polanyi potential, R (KJ mol^−1^ K^−1^ ) is the gas constant, T (K) is temperature, Xm (g g^−1^) is the adsorption capacity of the adsorbent, and k (mol^2^ KJ^−2^ ) is the DR isotherm constant related to the adsorption energy through the following equation:

E = 1/√(2k)(3)

The obtained values of the sorption parameters are reported in Table 4.

As for the dye, the *E* values obtained were in the range of adsorption energy (8–16 KJ mol^–1^) characteristic for adsorption systems dominated by chemical ion-exchange mechanism [122,123,124], while for the metal ions a borderline value was obtained, thus indicating that occurrence of other mechanisms than cation exchange, i.e., direct bonding between metal cations with the surface of clay (electrostatic interactions) and/or surface complexation [125].

The different modes of adsorption and their dependence on the pH conditions suggest the possibility to properly modulate the removal and recovery of effluent contaminants. Therefore, the employment of Mt nanoclay as sorbent offer a versatile method for the decontamination and valorization of wastewaters containing different types of pollutants. 

### 3.3. XRD Characterization

**Figure 3:**
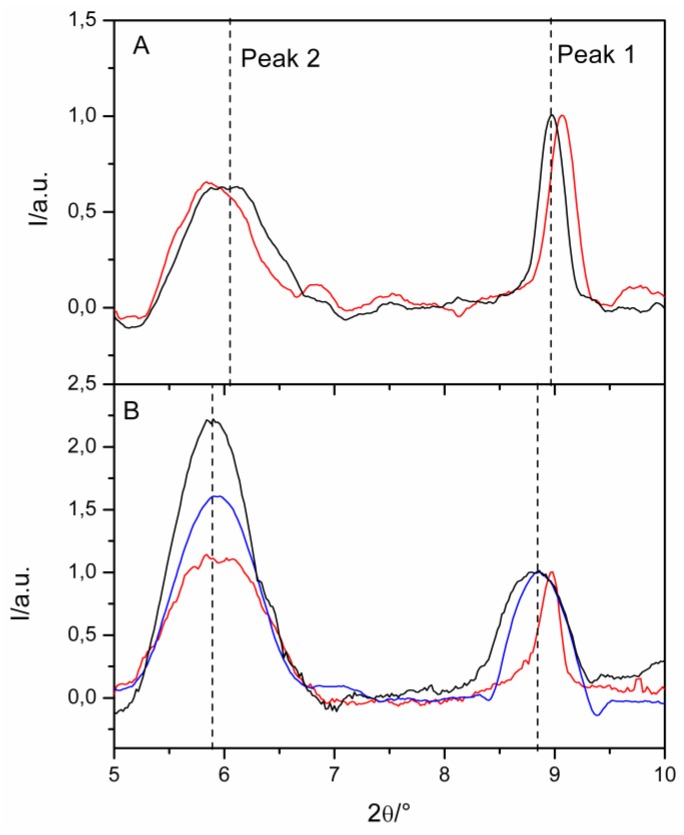
X-ray diffraction (XRD) patterns of pristine Mt (black line), CV/Mt hybrids (red line) and Ce(III)/Mt (blue line) at prepared at pH = 3.0 (**A**) and pH = 7.0 (**B**).

XRD patterns of unmodified and modified Mt samples, registered in the very low angle range, are reported in Figure 3.

Perusal of diffractograms evidences the presence of the peaks characteristic of the hydrated montmorillonite k10 clay, i.e., one at 2θ approximately equal to 8.9° (peak 1) which corresponds to the basal interlayer, and a reflection peak at a lower 2θ value (~6°) (peak 2), which, according to literature [74,126,127,128,129,130], is attributed to the majority of the interlayer spaces being intercalated with water molecules as proved by the disappearance of this refection peak after dehydration processes [129].

Comparison between the XRD patterns of the unmodified Mt hydrated at the pH 3.0 and 7.0, reveals that acidic conditions lead to a shrinking of the clay interlayer. This can probably be due to exchange processes between H^+^ and the larger cations placed in the clay structure.

Hybrid samples have structural characteristics nearly identical to the unmodified Mt, thus indicating that the clay structure is maintained during the adsorption processes.

As for the positions of the peaks, no changes are detected in the presence of the metal Ce(III), which means that the clay interlayer was not affected by Ce(III) exchange reactions. This behavior is consistent with the results of the adsorption isotherms previously described and it is in line with the study of [131] that suggested that metal cations were fixed solely on the outer surfaces of the clay.

By contrast, the adsorption of CV leads to a small shift in the peaks positions. In more detail, peak 1 moves towards higher 2θ values, at both investigated pH values, thus indicating a contraction of the basal interlayer. The lower interplanar distance after dye adsorption could be taken as an indication of the occurrence of cation exchange processes which displace cations from the interlayer spaces as already observed in (Bromberg et al., 2011; Calabrese et al., 2017; Cui et al., 2008). 

A perusal of the position of peak 2 reveals that the entrance of CV at pH 3.0 leads to an enlargement of the interlayer spaces intercalated with water molecules from d = 14.5 Å to d = 15.0 Å, while at pH 7.0 the dimension of the clay interlayer is already d = 15.0 Å and no changes are detected. 

It is worth to underline that the different behavior of metal and dye is in accordance with the results of the adsorption isotherms and it corroborates the hypothesis that the dye adsorbs onto Mt clay through both exchange processes and interaction with the outer surface, while metal ions do not enter the clay interlayer. 

### 3.4. Protocol for the Removal and Separation of Pollutants 

The information obtained about the different behavior of the two pollutants on varying pH was exploited to develop a procedure for the removal and separation of the two species from a solution mimicking an effluent containing the same amount of the two contaminants (~8 × 10^−2^ g dm^−3^).

**Figure 4:**
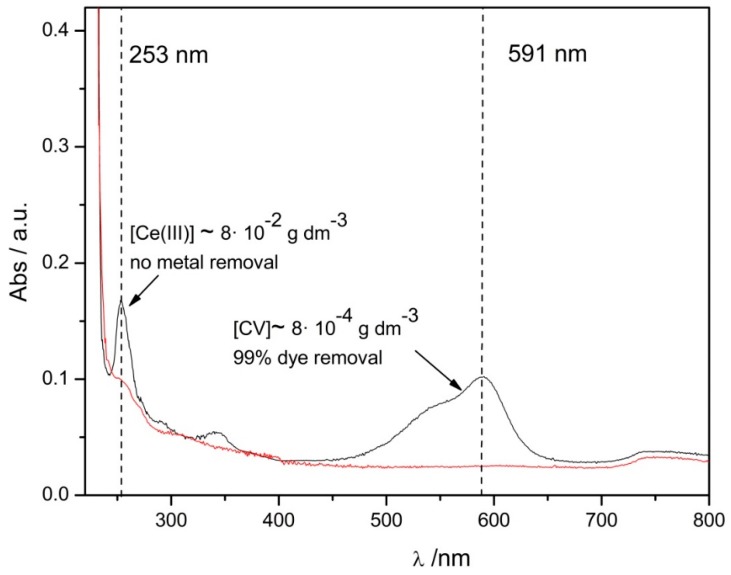
Ultraviolet–visible (UV–vis) spectrum of a mixture containing ~8 × 10^−2^ g dm^−3^ of CV and Ce(III) after the treatment with Mt at pH 3.0 (black line) and after the subsequent with Mt at pH 7.0 (red line).

The solution was treated, according to the method already developed in the first part of the present work, with a suspension of Mt (2.0 g dm^−3^) at pH 3.0 in order to remove only the CV. The ultraviolet–visible (UV–vis) spectrum of the supernatant obtained from the centrifugation of the obtained dispersion was registered (see Figure 4), then pH was brought to 7.0 and treated again with Mt, in order to remove the metal ions from the solution. 

The spectrum of the pollutant’s mixture after the treatment with Mt at pH 3.0 (black line) reveals the presence of a peak corresponding to ~8 × 10^−4^ g dm^−3^ of CV (λ = 591 nm) indicating the removal of the 99% of the dye, and a peak corresponding to ~8 × 10^−2^ g dm^−3^ of Ce(III) (λ = 253 nm) indicating that the applied procedure does not remove the metal from the solution at all. The subsequent treatment at pH 7.0 (red line) leads to the total removal of both the contaminants.

In the light of the results obtained it can be concluded that the proposed protocol can be efficiently applied for the separation and removal of the two different kinds of pollutant, if simultaneously present in a wastewater sample. Although, at this stage, experiments on the regeneration of the clay were not still performed, the results obtained open up the possibility to recover and re-use the two contaminants

Experiments were also performed where a solution containing the same amount of Ce(III) and CV (8 × 10^−2^ g dm^−3^) at pH 7.0 was treated with 2.0 g dm^−3^ of Mt. It was observed that the procedure allows the removal of both contaminants and can be applied efficiently when the separation of the contaminants is not required.

## 4. Conclusions

The performance of montmorillonite clay in the decontamination of aqueous solutions containing different types of contaminants were verified here. Crystal violet and Cerium (III) were chosen as models for dyes and metals. The adsorption process from effluents containing the two pollutants separately was first investigated at pH 3.0 and pH = 7.0, thus revealing significant differences in the behavior of the two species under the different experimental conditions. Adsorption isotherms and XRD measurements were performed in order to characterize the system. Then, based on the information obtained, a procedure was proposed and successfully applied to remove simultaneously and separate the metal ions and dye from wastewaters containing both contaminants.

These results can be helpful for further studies in scale-up processes using real effluents characterized by the presence of different types of pollutants. 

## Figures and Tables

**Table 1:** Molar adsorption coefficient values (ε, M^−1^ cm^−1^) of crystal violet (CV) and Ce(III) at pH 3.0 and 7.0.

	pH Conditions	ε, M^−1^ cm^−1^
**CV** **(λ _max_ = 591 nm)**	pH 3.0	42000 ± 800
	pH 7.0	73800 ± 300
**Ce(III)** **(λ _max_ = 253 nm)**	pH 3.0	740 ± 30
	pH 7.0	860 ± 50

**Table 2:** Weight percentage of CV and Ce(III) adsorbed onto Mt mineral clay.

	pH 3.0	pH 5.0	pH 7.0
**CV**	73 wt%	78 wt%	95 wt%
**Ce(III)**	0	0	50 wt%

**Table 3:** Sorption parameters and selected figures of merit of the two applied models, for the adsorption isotherms of the contaminants onto the Mt.

		**Langmuir** Cs=qmKLCe1+KLCe				
		**q^m^, g g^−1^**	**K_L_, dm^3^ g^−1^**	**R^2^**	**χ^2^**	**ESS**
**CV**	**pH 3.0**	0.155 ± 0.007	480 ± 60	0.969	6.0 × 10^−5^	1.3 × 10^−3^
	**pH 7.0**	0.22 ± 0.02	430 ± 70	0.961	8.8 × 10^−5^	1.0 × 10^−3^
**Ce(III)**	**pH 7.0**	0.122 ± 0.004	180 ± 20	0.984	1.0 × 10^−5^	9.2 × 10^−5^
		**Freundlich** $C_{s}=K_{F}C_{e}^{1/n}$				
		**n**	**K_F_, (g g^−1^) (dm^3^ g^−1^)^1/n^**	**R^2^**	**χ^2^**	**ESS**
**CV**	**pH 3.0**	2.2 ± 0.1	1.1 ± 0.1	0.985	2.9 × 10^−5^	6.3 × 10^−4^
	**pH 7.0**	2.0 ± 0.1	2.3 ± 0.4	0.967	7.4 × 10^−5^	8.8 × 10^−4^
**Ce(III)**	**pH 7.0**	2.5 ± 0.3	0.5 ± 0.1	0.923	5.0 × 10^−5^	4.5 × 10^−4^

**Table 4:** Sorption parameters of the Dubinin–Radushkevich model for the adsorption isotherms of the contaminants onto the Mt.

		X^m^, g g^−1^	K, mol^2^ KJ^−2^	E, KJ mol^−1^	R^2^
**CV**	**pH = 3.0**	0.47 ± 0.08	(5.9 ± 0.2) × 10^−4^	9.2 ± 0.3	0.9599
	**pH = 7.0**	0.29 ± 0.05	(6.7 ± 0.5) × 10^−4^	8.6 ± 0.6	0.9349
**Ce(III)**	**pH = 7.0**	0.23 ± 0.01	(8.8 ± 0.7) × 10^−4^	7.5 ± 0.6	0.9377
